# Clinical symptoms, diagnosis, treatment, and outcome of COVID‐19‐associated encephalitis: A systematic review of case reports and case series

**DOI:** 10.1002/jcla.24426

**Published:** 2022-04-18

**Authors:** Maryam Koupaei, Negar Shadab Mehr, Mohamad Hosein Mohamadi, Arezoo Asadi, Sajjad Abbasimoghaddam, Amirhosein Shekartabar, Mohsen Heidary, Fazlollah Shokri

**Affiliations:** ^1^ 48462 Department of Microbiology School of Medicine Kashan University of Medical Sciences Tehran Iran; ^2^ 56941 Student Research Committee Sabzevar University of Medical Sciences Sabzevar Iran; ^3^ Microbial Biotechnology Research Center Iran University of Medical Sciences Tehran Iran; ^4^ 440827 Department of Microbiology School of Medicine Iran University of Medical Sciences Tehran Iran; ^5^ 56941 Cellular and Molecular Research Center Sabzevar University of Medical Sciences Sabzevar Iran; ^6^ 56941 Department of Laboratory Sciences School of Paramedical Sciences Sabzevar University of Medical Sciences Sabzevar Iran; ^7^ 41616 Department of Medical Genetics Faculty of Medical Sciences Tarbiat Modares University Tehran Iran

**Keywords:** COVID‐19, encephalitis, SARS‐CoV‐2, systematic review

## Abstract

**Introduction:**

Since COVID‐19 outbreak, various studies mentioned the occurrence of neurological disorders. Of these, encephalitis is known as a critical neurological complication in COVID‐19 patients. Numerous case reports and case series have found encephalitis in relation to COVID‐19, which have not been systematically reviewed. This study aims to evaluate the clinical symptoms, diagnosis, treatment, and outcome of COVID‐19‐associated encephalitis.

**Methods:**

We used the Pubmed/Medline, Embase, and Web of Science databases to search for reports on COVID‐19‐associated encephalitis from January 1, 2019, to March 7, 2021. The irrelevant studies were excluded based on screening and further evaluation. Then, the information relating diagnosis, treatment, clinical manifestations, comorbidities, and outcome was extracted and evaluated.

**Results:**

From 4455 initial studies, 45 articles met our criteria and were selected for further evaluation. Included publications reported an overall number of 53 COVID‐19‐related encephalitis cases. MRI showed hyperintensity of brain regions including white matter (44.68%), temporal lobe (17.02%), and thalamus (12.76%). Also, brain CT scan revealed the hypodensity of the white matter (17.14%) and cerebral hemorrhages/hemorrhagic foci (11.42%) as the most frequent findings. The IV methylprednisolone/oral prednisone (36.11%), IV immunoglobulin (27.77%), and acyclovir (16.66%) were more preferred for COVID‐19 patients with encephalitis. From the 46 patients, 13 (28.26%) patients were died in the hospital.

**Conclusion:**

In this systematic review, characteristics of COVID‐19‐associated encephalitis including clinical symptoms, diagnosis, treatment, and outcome were described. COVID‐19‐associated encephalitis can accompany with other neurological symptoms and involve different brain. Although majority of encephalitis condition are reversible, but it can lead to life‐threatening status. Therefore, further investigation of COVID‐19‐associated encephalitis is required.

## Graphical abstract

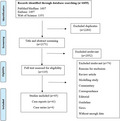

## 1 INTRODUCTION

Humans have been struggling with the COVID‐19 epidemic for nearly 2 years.^1^ As of early August 2020, more than 17.5 million cases of COVID‐19 were identified in 188 countries, including 680,000 deaths.^2^ The disease that often has respiratory symptoms but sometimes also has extrapulmonary manifestations such as neurological symptoms.^1^ On average, neurological symptoms appear three weeks after respiratory symptoms.^3^ Less common clinical manifestations of COVID‐19 include headache, brain status alteration, chest pain, abdominal pain, diarrhea, and nausea.^2^ Nervous manifestations can range from a mild nervous agitation to severe encephalitis.^4^ The roll of central nervous system in SARS‐CoV‐2 epidemic has been determined.^3^ Ischemic stroke, central nervous system (CNS) inflammation, encephalopathy, and myelitis are common clinical manifestations of the CNS in COVID‐19 patients.^5^ Encephalitis means inflammation of the brain,^6^ which is mainly caused by the autoimmune process and/or the viral infection.^5^ Encephalitis is one of the main and devastating complications associated with CNS.^7^ In previous epidemics, MERS‐CoV and SARS‐CoV viruses have caused brain complications such as polyneuropathy, ischemic stroke, encephalitis, and brain status change in patients with Middle East respiratory syndrome coronavirus and severe acute respiratory syndrome coronavirus.^8^, ^9^ Several case reports and case series have reported the patients with COVID‐19‐associated encephalitis, which in some cases have been fatal.^4^, ^10^, ^11^ The pooled mortality rate from COVID‐19‐associated encephalitis is reported to be 13.4%.^12^ In a multicenter study by Pilotto et al. in Italy, 25 out of 45 people with encephalitis tested positive for SARS‐CoV‐2. They found that there is a wide range of clinical manifestations in patients and the response to treatment depends on the specific CNS manifestations.^13^ Due to the importance of encephalitis in COVID‐19 patients and the risk of death for them, it is necessary to conduct a detailed systematic review on this study. Therefore, the aim of this study was to evaluate the clinical symptoms, diagnosis, treatment, and outcome of COVID‐19‐associated encephalitis.

## 2 MATERIALS AND METHODS

This systematic review was performed according to “Preferred Reporting Items for Systematic Reviews and Meta‐Analyses” (PRISMA) statement.^14^

### 2.1 Search strategy

We used Pubmed/Medline, Embase, and Web of Science databases for this literature. The articles included were only those published in English from January 1, 2019, to March 7, 2021. The search keywords used were “encephalitis,” “brain,” “neurologic,” “COVID‐19,” “severe acute respiratory syndrome coronavirus 2,” “SARS‐CoV‐2,” “2019‐nCoV,” “nCoV disease,” “coronavirus disease‐19,” “2019 novel coronavirus,” and “Wuhan pneumonia.”

### 2.2 Inclusion/exclusion criteria

Case reports and case series reporting encephalitis in patients with COVID‐19 were included. These studies met the following inclusion criteria: (i) COVID‐19 patients were confirmed and diagnosed with RT‐PCR as suggested by WHO; (ii) the raw data for clinical symptoms, diagnosis, treatment, and outcome of COVID‐19‐associated encephalitis were addressed. Studies without enough data, review article, modelling study, commentary, correspondence, editorial, guideline, and news were excluded. All potentially relevant articles were then screened for eligibility. Two reviewers independently screened the records by title, abstract, and full texts to exclude those not related to the current study.

### 2.3 Data collection

The extracted data included first author name; country where the study was conducted; year of publication; type of study; number of patients investigated; distribution of age and sex in the population; diagnosis methods; data for clinical, radiological, and laboratory findings; therapy, and the patient outcome.

### 2.4 Quality assessment

We used the case reports/case series appraisal checklist supplied by the Joanna Briggs Institute (JBI) to evaluate the quality of the studies.^15^

## 3 RESULTS

### 3.1 Study selection and general characteristics

**FIGURE 1:**
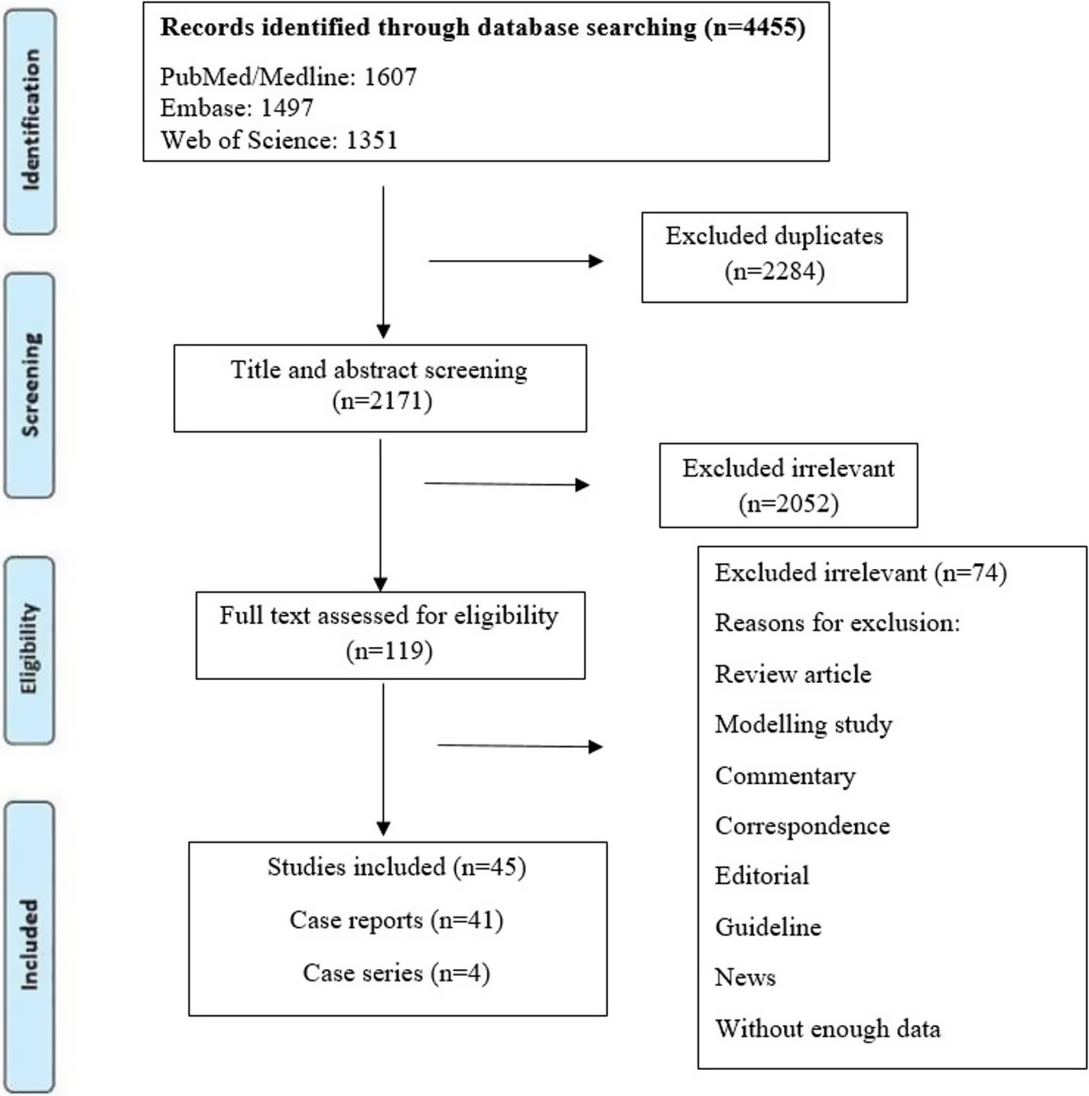
Flow diagram detailing review process and study selection

As shown in Figure 1, at the first screening, 4455 papers were retrieved. In the second phase, after removing duplicates, 2171 papers remained. These papers were screened by title and abstract, and 119 were selected for detailed full‐text evaluation. Applying the criteria to the full‐text documents, 45 articles were eligible for inclusion in the systematic review. The results of various studies including participants’ clinical manifestations, comorbidities, diagnosis, treatment, and outcome are reported in Tables 1 and 2. Moreover, a summary of the case report and case series findings are reported in Table 3.

**TABLE 1:** Characteristics of the case series studies

First author	Country	Published time	No. of patients	Median age (years)	Male/female	Encephalitis diagnosis method	CT results	MRI results	Special encephalitis treatment	SARS‐CoV‐2 diagnosis method	COVID‐19 treatment	Clinical manifestations	SARS‐CoV‐2 diagnosis in CSF sample	Comorbidities	Outcomes
Lopes CCB^32^	Brazil	December 2020	2	50	1M, 1F	2 brain CT scan, 2 brain MRI, 2 EEG	2 bilateral lesions in the centrum semiovale/1 focal lesions in globus pallidus and internal capsule/1 signal abnormalities in the white matter, including corpus callosum	2 multifocal hyperintensity in centrum semiovale, 1 lesions in the cerebellar white matter and globus pallidus	NM	2 RT‐PCR	1 hydroxychloroquine	2 fever, 2 RS, 1 renal failure, 2 delayed awakening after sedation withdrawal, 2 DC, 1 coma, 1 four‐limb weakness	2 negative	2 hypertension, 1 diabetes, 1 obesity, 1 smoking	1 death, 1 partial recovery
Kihira S^33^	USA	October 2020	5	48.6	3M, 2F	MRI, head CT, EEG	5 unremarkable	5 hyperintensity in the white matter, 3 confluent diffusion restriction in the cerebral white matter, 2 hyperintensity in the splenium of corpus callosum/1 scattered frontoparietal hyperintensity/1 microhemorrhages in corpus callosum/1 intraventricular hemorrhage	1 plasma exchange	5 RT‐PCR	NM	1 myocardial infarction, 2 fever, 5 RS, 3 acute renal failure, 1 cardiogenic shock, 1 abdominal pain, 1 cardiac arrest, 1 nausea, 1 vomiting, 1 chills, 5 AMS, 1 focal seizures, 1 lower limbs paralysis	3 negative, 1 NP, 1 NM	1 Hypertension, 1 diabetes mellitus type 2, 1 pyelonephritis, 1 gestation, 1 obesity	NM
Barreto‐Acevedo E^34^	Peru	June, 2020	2	50.5	1M, 1F	MRI, CSF analysis, brain tomography	2 unremarkable	1unremarkable, 1 NP	Dexamethasone	RT‐PCR, serological test	NM	2 fever, 2 chills, 1 malaise, 1 headache, 2 AMS, 2 seizures	NP	1 obesity	1death, 1 transferred to other hospital
Delorme C^35^	France	August 2020	4	66.75	2M, 2F	Brain MRI, CSF analysis, brain FDG‐PET/CT imaging, EEG	4 NP	1 unremarkable/1 non‐specific Hyperintensity of the white matter/1 right T2 orbitofrontal Hyperintensity	3 IV immunoglobulin, 3 IV corticosteroids	4 RT‐PCR	NM	4 cognitive impairment, 2 cerebellar syndrome, 1 myoclonus, 1 psychiatric symptoms, 4 fever, 3 RS, 2 anosmia, 1 ageusia, 1 diarrhea, 2 fatigue, 2 agitation, 1 psychomotor slowing, 1 convulsive status, 1 apraxia, 1 dysexecutive syndrome	4 negative	1 temporal lobe epilepsy (hippocampal sclerosis), 1 diabetes mellitus type2, 1 hypertension	4 discharged

**TABLE 2:** Characteristics of case report studies

First author	Country	Published time	Age (years)	Sex	Encephalitis diagnosis method	CT results	MRI results	Special encephalitis treatment	SARS‐CoV‐2 diagnosis method	COVID‐19 treatment	Clinical manifestations	SARS‐CoV‐2 diagnosis in CSF sample	Comorbidities	Outcomes
Kumar N^36^	India	October 2020	35	M	Head CT	Hypodensities in both thalami and left caudate nucleus	NP	Propofol infusion, mannitol, IV methylprednisolone	RT‐PCR	Hydroxychloroquine, azithromycin, IV amoxicillin‐clavulanic acid	Fever, vomiting, DC	NP	Invasive meningioma	Death
Novi G^37^	Italy	September 2020	64	F	Brain and spine MRI, CSF analysis	NP	Multiple enhancing lesions of the brain, bilateral optic nerve enhancement	IV methylprednisolone, prednisone	RT‐PCR, antibody testing	IV immunoglobulin	RS, anosmia, ageusia, visual impairment, behavioral changes, headache, hyperreflexia	Positive	Vitiligo, hypertension, monoclonal gammopathy	Discharged
Ayuso LL^38^	Spain	September 2020	72	F	Brain MRI, immunoblot analysis	NP	Hyperintensity in cerebellum, contrast enhancement on the floor of the fourth ventricle	IV methylprednisolone, prednisone	RT‐PCR, chest CT	Hydroxychloroquine, azithromycin, ceftriaxone	Psychiatric symptoms, fever, AMS, dizziness, visual impairment, unsteadiness, cerebellar signs	NP	Hypertension, hyperlipidemia, smoking, depression	Discharged
Khan Z^39^	USA	November 2020	30	M	Head CT	Opacifications of paranasal sinuses, hypodensity of the white matter	Hyperintensity in the white matter of cerebral hemispheres	Acyclovir	RT‐PCR, chest CTy	NM	Seizure, DC, behavioral changes, myoclonus, AMS, psychiatric symptoms	NP	Obesity	Still hospitalized
Westhoff TH^40^	Germany	July 2020	69	M	Brain MRI, CSF analysis	NP	Linear meningeal enhancement/hyperintensity in the white matter	NM	RT‐PCR, chest CT	Hydroxychloroquine	Fever, RS, diarrhea, pancreas and kidney allograft dysfunction, seizure, hemi‐neglect, fatigue	Positive	Immunosuppression	Discharged
Kamal YM^41^	United Arab Emirate	September 2020	31	M	CSF analysis, head CT, brain MRI	Bilateral hypodensities in the external capsules, the insular cortex and white matter of the frontal lobes	Bilateral diffusion restriction in the temporal and frontal lobes/bilateral hyperintensity in the temporal lobe cortex	IV acyclovir sodium	RT‐PCR	Chloroquine, lopinavir–ritonavir	Behavioral changes, AMS, agitation, drowsiness	Positive	None	Discharged
Rebeiz T^21^	USA	September 2020	NM	M	CSF analysis, brain MRI	Subarachnoid hemorrhage within the mesial parietal region/nonspecific hypo‐attenuation in the splenium of the corpus callosum	Diffusion restriction and hyperintensity of the corpus callosum, left thalamus and frontal cortex	Acyclovir, ceftriaxone, vancomycin	RT‐PCR	NM	Behavioral changes, fever, AMS, psychiatric symptoms	NP	None	Death
Zoghi A^42^	Iran	June 2020	21	M	CSF analysis, brain and cervical MRI	NP	Hyperintensity in the internal capsule, cerebral peduncles, pons and the corpus callosum	IV vancomycin, meropenem, acyclovir	Chest CT, antibody testing	Plasma exchange	Anorexia, vomiting, food intolerance, malaise, lower limbs paralysis, weakness, urinary retention, drowsiness	Negative	None	NM
Moriguchi T^9^	Japan	May 2020	24	M	Brain MRI	NP	Hyperintensity along the wall of right lateral ventricle, right temporal lobe and hippocampus, slight hippocampus atrophy	IV ceftriaxone, vancomycin, acyclovir, steroids	Chest CT, RT‐PCR	Favipiravir	Fatigue, fever, headache, RS, seizure, unconsciousness	Positive	NM	Still hospitalized
Haqiqi A^43^	United kingdom	January 2021	56	M	Head CT, brain MRI, CSF analysis	Diffuse hypodensity of the white matter/multiple bilateral white matter hemorrhagic foci involving the corpus callosum	Hyperintensity of the white matter/diffuse hemosiderin staining throughout the white matter and the corpus callosum/some cystic hemorrhagic areas within both cerebral hemispheres	None	RT‐PCR	Supportive care	RS, acute kidney injury, DC	Negative	Hypertension, chronic kidney disease, hypercholesterolemia, asthma, obesity	Discharged
Pizzanelli C^44^	Italy	January 2021	74	F	Brain MRI, total body PET/TC	Unremarkable	Hyperintensity in the temporal lobes, mild hippocampal thickening	IV methylprednisolone, oral prednisolone	Chest CT, RT‐PCR	Remdesevir, dexamethasone	Fever, RS, seizure, AMS, oral automatism, weakness, ideo‐motor slowing	Negative	None	Discharged
Al Mazrouei SS^1^	United Arab Emirates	September 2020	43	M	Head CT, brain MRI	Hypodensity of bilateral thalami	Hyperintensity in the frontal lobes, insula, thalamus and globus pallidus	NM	RT‐PCR, Chest CT	NM	Fever, RS, weakness, fatigue, DC	NP	Diabetes mellitus type2	Death
Sirous R^2^	USA	August 2020	50	M	MRI, magnetic resonance angiography, magnetic resonance venography	Mild cerebral generalized parenchymal volume loss with sulcal enlargement	Cerebral edema with mass effect, downward cerebellar tonsillar herniation/compression and displacement of the brainstem and 4th ventricle	NM	RT‐PCR	IV hydroxychloroquine	Fatigue, headache, nausea, vomiting, lethargy, AMS	NP	None	Death
Mardani M^45^	Iran	July 2020	64	F	CSF analysis	Unremarkable	NP	NM	RT‐PCR, CSF analysis	IV ceftriaxone, clindamycin, hydroxychloroquine, lopinavir/ritonavir	RS, weakness, DC	Positive	Hypertension, ischemic heart disease, metastatic colorectal cancer	NM
Vandervorst F^46^	Belgium	July 2020	29	M	Brain MRI	Unremarkable	Hyperintensity of the left temporal cortex/mild gyral expansion	NM	RT‐PCR, chest CT	IV acyclovir, hydroxychloroquine	Weakness, RS, anorexia, Anosmia, ageusia, AMS, short‐term memory deficits, psychiatric symptoms	Negative	None	Discharged
Freire‐Álvarez E^31^	Spain	October 2020	39	M	Brain MRI	Unremarkable	Hyperintensity at the cortical and subcortical right frontal regions, right thalamus and mammalary body, temporal lobes and cerebral peduncles	IV immunoglobulin, tocilizumab	RT‐PCR, chest CT	Lopinavir/ritonavir, subcutaneous interferon beta‐1b	Fatigue, DC, malaise, fever, AMS, headache, drowsiness, minimal stiff neck, language disorder, paraphasia	Negative	NM	Clinical improvement, Still hospitalized
Parsons T^47^	Germany	May 2020	51	F	Brain MRI, EEG	NP	Hyperintensities in the white matter	Methylprednisolone, IV Immunoglobulin	RT‐PCR, chest CT	NM	RS, fever, vomiting, unresponsiveness, flaccid muscles	Negative	NM	NM
Al‐olama M^48^	United Arab Emirates	May 2020	36	M	Brain CT, CT angiography	Hematoma in the right frontal lobe with surrounding edema/extracerebral hemorrhage/cortical swelling/bilateral supratentorial leptomeningeal increased enhancement	NP	NM	PCR	NM	Fever, RS, headache, body pain, diarrhea, vomiting, drowsiness, AMS	NP	None	Still hospitalized
Goodloe TB^49^	Alabama	January, 2021	52	M	Bead CT, EEG	Unremarkable	Unremarkable	Vancomycin, ceftriaxone, azithromycin, acyclovir	RT‐PCR	NM	AMS, agitation, fever	NP	Hypertension, diabetes mellitus type2, end‐stage renal disease, coronary artery disease	Discharged
Sattar SBA^23^	USA	September 2020	44	M	Brain MRI, head CT, CSF analysis	Few scattered foci of white matter hypo‐attenuation	Abnormal medial cortical signals in the bilateral frontal lobes	NM	RT‐PCR, chest CT	Hydroxychloroquine, azithromycin	Fever, RS, seizure, AMS, unresponsiveness	Positive	None	Discharged
Haider A^29^	USA	March 2020	66	M	EEG, brain MRI	Unremarkable	Small lacunar infarcts and a patchy area of bright signals in the cortical and lateral periventricular regions	Tocilizumab, IV immunoglobulin, rituximab	RT‐PCR	NM	Seizure, AMS, behavioral changes	NP	Benign prostatic hypertrophy, fatty liver disease, hypertension	Discharged
Cariddi LP^50^	Italy	June, 2020	64	F	Head CT, brain MRI	Bilateral hypodensity of the white matter/a small left occipital parenchymal hemorrhage	Bilateral edema with bilateral occipital foci of subacute hemorrhage	NM	RT‐PCR	Hydroxychloroquine, darunavir/cobicistat	Fever, RS, visual impairment, AMS, drowsiness, reduced tendon reflexes	Negative	Hypertension, gastroesophageal reflux disease, hyperuricemia, dyslipidemia, obstructive sleep apnea, atrial fibrillation	Partial recovery
Sofijanova A^51^	Republic of Macedonia	November 2020	9 month	NM	Head CT, biochemical blood test	Enlargement of the lateral ventricles, with intraventricular masses, internal hydrocephalus	NP	Anti‐edematous therapy	NM	Cephalosporin, aminoglycoside, antiviral drug	RS, convulsive status, fever, DC, vomiting, seizure	NP	NM	Transferred to another hospital
Ghosh R^11^	India	August 2020	44	F	Brain MRI, CSF analysis	NP	T2‐weighted hyperintensity in the parietal lobes with peri‐lesional edema	IV methylprednisolone	RT‐PCR	Ceftriaxone, vancomycin, acyclovir	Myalgia, RS, hypogeusia, hyposmia, AMS, seizure, unconsciousness, reduced tendon refluxes, loss of sphincter control	NP	None	Death
Pilotto A^52^	Italy	August, 2020	60	M	EEG, brain MRI, CSF analysis	Unremarkable	Unremarkable	Methylprednisolone	RT‐PCR, chest CT	Ritonavir/lopinavir, hydroxychlroroquine	Fever, RS, cognitive fluctuations, DC, AMS, behavioral changes, asthenia	Negative	None	Discharged
Azab MA^3^	Egypt	February, 2021	89	M	MRI, post‐mortem biopsy	NP	Hyperintensity near the basal ganglia and thalami	NM	Serological test	Acyclovir, acetaminophen	Rash, seizure, tremors, RS, cerebellar signs, fever, headache, dizziness, myalgia	NP	NM	Death
Abdi S^53^	Iran	June, 2020	58	M	Brain MRI, CSF analysis	NP	Hyperintensity of the white matter/involvement of cortical and deep gray matter and midbrain	IV dexamethasone	RT‐PCR, chest CT	NM	Drowsiness, gait disturbance, DC	Negative	NM	Death
Dharsandiya M^54^	India	August, 2020	68	M	Head CT, blood test, CSF analysis	Age‐related cortical atrophy (unremarkable)	NP	Methylprednisolone, tocilizumab	RT‐PCR, chest CT	Azithromycin, hydroxychloroquine, gamma globulin	Fever, RS, renal failure, viral sepsis, autonomic disturbance, AMS, seizure	NP	Diabetes, hypertension	Death
Babar A^55^	USA	October, 2020	20	F	Brain MRI, CSF analysis, EEG	Unremarkable	Unremarkable	Methylprednisolone	RT‐PCR	Levofloxacin, acyclovir	RS, ageusia, insomnia, Fever, AMS, psychiatric symptoms	Negative	Obesity, anxiety	Discharged
Virhammar J^56^	Sweden	June, 2020	55	F	Head CT, CSF analysis, EEG, brain MRI	Hypodensities in the thalami and midbrain	Hyperintensity in subinsular regions, thalami, and brainstem/involvement of temporal lobes, hippocampi, and cerebral peduncles	IV immunoglobulin	RT‐PCR, chest CT	Acyclovir, plasma exchange	Fever, myalgia, impaired brain stem reflexes, myoclonus, lethargy, DC	Positive	None	Discharged to rehabilitation
Farhadian S^57^	USA	June, 2020	78	F	Brain MRI, EEG, CSF analysis	NP	Generalized atrophy/hyperintensity in white matter	NM	RT‐PCR, chest CT	Hydroxychloroquine	Seizure like activity, RS, fever, AMS	Negative	Immunosuppression due to kidney transplantation	Discharged
de Miranda Henriques‐Souza AM^58^	Brazil	October, 2020	12	F	Brain and spine MRI, CSF analysis	NP	Bilateral restricted diffusion in the white matter/hyperintensity of the corpus callosum	Methylprednisolone	RT‐PCR	NM	Tetraplegia, fever, deep areflexia, skin rash, headache, RS, acute motor weakness, numbness	Negative	None	Discharged
Afshar H^59^	Iran	August 2020	39	F	Brain MRI	NP	Hyperintensities in bilateral thalami, temporal lobes and pons	IV immunoglobulin, IV methylprednisolone	Chest CT	Meropene, levofloxacin, linezolide, hydroxychloroqine, atazanavir, IV immunoglobulin	Fever, myalgia, anorexia, drowsiness, RS, DC, headache, seizure	Negative	None	Discharged
Crosta F^60^	Italy	December 2020	79	M	EEG, brain MRI	Unremarkable	Hyperintensity of the left temporal cortex, with mild gyral expansion	NM	RT‐PCR	Clarithromycin, dexamethasone	Fever, AMS, anosmia, RS, ageusia, DC, short‐term memory deficits, psychiatric symptoms	NP	Hypertension, diabetes, chronic heart failure	Discharged
Sangare A^61^	France	November 2020	56	M	EEG, brain MRI	NP	Hemorrhagic lesions in the pontine tegmentum and subinsular regions, including corpus callosum	IV methylprednisolone, plasma exchange with albumin	RT‐PCR, chest CT	Cephalosporin linezolide, trimethoprime‐sulfamethoxazole, meropenem aminosid	Fever, RS, reversible acute kidney failure, visual impairment, unresponsiveness	Negative	Hypertension	Discharged
El‐Zein RS^62^	USA	September 2020	40	M	EEG, blood tests, CSF analysis	Unremarkable	Unremarkable	IV immunoglobulin	Simplexa SARS‐CoV‐2 assay	Hydroxychloroquine	Fever, fatigue, AMS, RS, psychiatric symptoms, increased agitation	Negative	None	Discharged
Etemadifar M^4^	Iran	September 2020	51	M	Head CT, brain MRI	Generalized brain edema/signs of brain herniation	Generalized brain edema, downward herniation of cerebellar tonsils and brainstem, hyperintensities in bilateral cerebral cortices and corpus striatum	NM	RT‐PCR	Hydroxychloroquine, lopinavir/ritonavir, IV acyclovir, IV dexamethasone	Headache, drowsiness, nausea, vomiting, RS, seizure, cardiac arrest, impaired brain stem reflexes	NP	Hypothyroidism migraine	Death
Peng LV^63^	China	February, 2021	90	F	CSF analysis, physical and neurological examination	Unremarkable	NP	Mannitol and anti‑viral therapy (Ganciclovir)	RT‑PCR, Chest CT	NM	Fever, RS, fatigue, unconsciousness, unresponsiveness, increased muscle tension	Negative	Cerebral lacunar infarction with no neurological deficits‐ live in a healthcare unit	Death (irrelevant cause)
Hayashi M^10^	Japan	August 2020	75	M	Neurological examination, brain MRI	NP	Hyperintensity in the splenium of corpus callosum	Corticosteroid pulse, meropenem	RT‐PCR	Favipiravir, corticosteroid pulse	Urinary incontinence, diarrhea, DC, cerebellar signs, fever, AMS, tremor, gait disturbance	NP	Mild Alzheimer's disease	Death
Kumar A^64^	USA	November 2020	35	F	Brain MRI, EEG, CSF analysis	Unremarkable	Hyperintensity in the white matter involving bilateral cerebral peduncles	Methylprednisolone, IV immunoglobulin, plasma exchange	RT‐PCR, serological tests	NM	Anosmia, ageusia, gait disturbance, neuropathy, weakness, drowsiness, lethargy	Negative	Gastric bypass surgery, anemia	Discharged to a long‐term care facility
Muccioli L^65^	Italy	September 2020	47	F	EEG, brain MRI	NP	Hyperintensity in the white matter	Tocilizumab	Chest CT, RT‐PCR	NM	Asthenia, RS, ageusia, hyposmia, language disturbance, pain in the extremities, fever, AMS, headache, agitation	Negative	None	Discharged

**TABLE 3:** Summary of the case reports and case series findings

Variables	No. of studies	*n*/*N*	%
Gender			
Male	29	32/53	60.38
Female	19	21/53	39.62
Age			
<30 (years old)	6	6/53	11.32
31–50 (years old)	16	18/53	33.96
>51 (years old)	25	29/53	54.72
Age/sex			
<30 (years old)			
Male	4	4/6	66.67
Female	2	2/6	33.33
31–50 (years old)			
Male	11	12/18	66.67
Female	6	6/18	33.33
>51 (years old)			
Male	15	16/29	55.17
Female	12	13/29	44.83
Clinical manifestation			
Neurological manifestations			
Decreased consciousness/unconsciousness	17	18/54	33.33
Behavioral changes	6	6/54	11.11
Altered mental status	24	29/54	53.70
Cerebellar signs	4	5/54	9.25
Seizure	15	16/54	29.62
Agitation	5	6/54	11.11
Headache	11	11/54	20.37
Memory deficits	2	2/54	3.70
Unresponsiveness	4	4/54	7.40
Convulsive status	2	2/54	3.70
Cognitive impairment	2	5/54	9.26
Language disturbance	2	2/54	3.70
Paraphasia	1	1/54	1.85
Tremors	2	2/54	3.70
Lower limbs paralysis	2	2/54	3.70
Gait disturbance	3	3/54	5.55
Unsteadiness	1	1/54	1.85
Hemi‐neglect	1	1/54	1.85
Impaired brain stem reflexes	2	2/54	3.70
Pain	3	3/54	5.55
Coma	1	1/54	1.85
Apraxia	1	1/54	1.85
Dysexecutive syndrome	1	1/54	1.85
Psychomotor slowing	1	1/54	1.85
Ideo‐motor slowing	1	1/54	1.85
Oral automatism	1	1/54	1.85
Neuropathy	1	1/54	1.85
Reduced tendon reflexes	2	2/54	3.70
Loss of sphincter control	1	1/54	1.85
Deep areflexia	1	1/54	1.85
Psychiatric symptoms			
Psychiatric symptoms	8	8/54	14.81
General symptoms			
Fever	32	38/54	70.37
Vomiting	8	8/54	14.81
Nausea	3	3/54	5.55
Diarrhea	4	4/54	7.40
Anosmia/hyposmia	7	8/54	14.81
Ageusia/dysgeusia	8	8/54	14.81
Dizziness	2	2/54	3.70
Malaise	3	3/54	5.55
Fatigue	8	9/54	16.66
Drowsiness	9	9/54	16.66
Weakness/asthenia	10	10/54	18.51
Lethargy	3	3/54	5.55
Chills	2	3/54	5.55
Anorexia	3	3/54	5.55
Food intolerance	1	1/54	1.85
Insomnia	1	1/54	1.85
Numbness	1	1/54	1.85
Neuromuscular symptoms			
Myalgia	4	4/54	7.40
Hyperreflexia	1	1/54	1.85
Myoclonus	3	3/54	5.55
Neck stiffness	1	1/54	1.85
Flaccid muscles	1	1/54	1.85
Tetraplegia	1	1/54	1.85
Increased muscle tension	1	1/54	1.85
Other			
Respiratory symptoms	30	37/54	68.51
Visual impairment	4	4/54	7.40
Renal dysfunction	8	10/54	18.51
Cardiac dysfunction	2	4/54	7.40
Rash	2	2/54	3.70
Viral sepsis	1	1/54	1.85
Delayed awakening after sedation	1	2/54	3.70
Autonomic disturbances	1	1/54	1.85
Comorbidities			
Hypertension	13	14/48	29.16
Diabetes mellitus	7	7/48	14.58
Obesity	6	6/48	12.50
Neurologic disorders	5	5/48	10.41
Cardiologic disorder	4	4/48	8.33
Dyslipidemia	2	2/48	4.16
Anemia	1	1/48	2.08
Psychiatric disorders	2	2/48	4.16
Renal dysfunction	3	3/48	6.25
Immunosuppressive state	2	2/48	4.16
Smoking	2	2/48	4.16
Hypercholesterolemia	1	1/48	2.08
Hypothyroidism	1	1/48	2.08
Vitiligo	1	1/48	2.08
Monoclonal gammopathy	1	1/48	2.08
Asthma	1	1/48	2.08
Colorectal cancer	1	1/48	2.08
Fatty liver disease	1	1/48	2.08
Gastroesophageal reflux disease	1	1/48	2.08
Hyperuricemia	1	1/48	2.08
Obstructive sleep apnea	1	1/48	2.08
Benign prostatic hypertrophy	1	1/28	3.57
Gestation	1	1/20	5.00
No comorbidities	15	15/48	31.25
Presence of SARS‐CoV‐2 RNA in the CSF sample			
Positive	7	7/34	20.58
Negative	21	27/34	79.41
SARS‐CoV‐2 diagnosis method			
RT‐PCR	40	49/53	92.45
Chest CT	20	20/53	37.73
Serological testing (anti‐SARS‐CoV‐2 antibody)	5	6/53	11.32
Simplexa SARS‐CoV‐2 assay	1	1/53	1.88
Encephalitis diagnosis method			
Brain MRI	36	44/54	81.48
Head CT scan	15	20/54	37.03
CSF analysis	21	25/54	46.29
Electroencephalogram	15	23/54	42.59
Total body PET/TC	1	1/54	1.85
FDG‐PET/CT imaging	1	4/54	7.40
CT angiogram	1	1/54	1.85
Magnetic resonance angiography and venography	1	1/54	1.85
Biochemical blood tests	3	3/54	5.55
Post‐mortem biopsy	1	1/54	1.85
Physical and neurological examination	2	2/54	3.70
Immunoblot analysis	1	1/54	1.85
Brain tomography	1	1/54	1.85
Special encephalitis treatment			
Dexamethasone	2	3/36	8.33
Plasma exchange	3	3/36	8.33
IV methylprednisolone/oral prednisone	13	13/36	36.11
IV immunoglobulin	8	10/36	27.77
Corticosteroids	2	4/36	11.11
Steroids	1	1/36	2.77
Propofol infusion	1	1/36	2.77
Mannitol	2	2/36	5.55
Acyclovir	6	6/36	16.66
Ceftriaxone	3	3/36	8.33
Vancomycin	4	4/36	11.11
Meropenem	2	2/36	5.55
Tocilizumab	4	4/36	11.11
Azithromycin	1	1/36	2.77
Rituximab	1	1/36	2.77
Anti‐edematous therapy	1	1/36	2.77
COVID‐19 treatment			
Hydroxychloroquine	15	15/30	50.00
Chloroquine	1	1/30	3.33
Azithromycin	4	4/30	13.33
IV amoxicillin‐clavulanic acid	1	1/30	3.33
IV immunoglobulin	2	2/30	6.66
Ceftriaxone	3	3/30	10.00
Dexamethasone	3	3/30	10.00
Favipiravir	2	2/30	6.66
Ritonavir/lopinavir	5	5/30	16.66
Plasma exchange	2	2/30	6.66
Remdesevir	1	1/30	3.33
Clarithromycin	1	1/30	3.33
Corticosteroid pulse	1	1/30	3.33
Clindamycin	1	1/30	3.33
Interferon beta‐1b	1	1/30	3.33
Darunavir/cobicistat	1	1/30	3.33
Cephalosporin	2	2/30	6.66
Aminoglycoside	1	1/30	3.33
Vancomycin	1	1/30	3.33
Linezolide	2	2/30	6.66
Acyclovir	6	6/30	20.00
Acetaminophen	1	1/30	3.33
Gamma globulin	1	1/30	3.33
Levofloxacin	2	2/30	6.66
Meropene	1	1/30	3.33
Atazanavir	1	1/30	3.33
Trimethoprime‐sulfamethoxazole	1	1/30	3.33
Meropenem aminosid	1	1/30	3.33
Outcome			
Death	13	13/46	28.26
Discharged	20	23/46	50.00
Discharged to rehabilitation/partial recovery	4	4/46	8.69
Still hospitalized	4	4/46	8.69
Transferred to another hospital	2	2/46	4.34
Brain MRI pattern			
Unremarkable	6	6/47	12.76
Hyperintensity in the white matter	15	21/47	44.68
Hyperintensity in the corpus callosum	5	6/47	12.76
Hyperintensity in the cerebellum	3	3/47	6.38
Hyperintensity of the thalamus	6	6/47	12.76
Hyperintensity in the temporal lobe	8	8/47	17.02
Hyperintensity in the frontal lobe	5	5/47	10.63
Hyperintensity in the brainstem	3	3/47	6.38
Hyperintensity in the parietal lobe	2	2/47	4.25
Hyperintensity along the wall of lateral ventricle	1	1/47	2.12
Hemorrhagic/microhemrorrhagic areas	4	5/47	10.63
Signs of brain edema	4	4/47	8.51
Confluent diffusion restriction in the white matter	2	4/47	8.51
Compression and displacement of the brainstem and fourth ventricle	1	1/47	2.12
Downward cerebellar tonsilar herniation	2	2/47	4.25
Mild gyral expansion	2	2/47	4.25
Involvement of cortical and deep gray matter and midbrain	1	1/47	2.12
Diffuse hemosiderin staining throughout the white matter and corpus callosum	1	1/47	2.12
Linear meningeal enhancement	1	1/47	2.12
Contrast enhancement on the floor of the fourth ventricle	1	1/47	2.12
Bilateral optic nerve enhancement	1	1/47	2.12
Slight hippocampus atrophy	1	1/47	2.12
Mild hippocampal thickening	1	1/47	2.12
Generalized brain atrophy	1	1/47	2.12
Head CT scan pattern			
Unremarkable	15	20/35	57.14
Hypodensity of the white matter	6	6/35	17.14
Hypodensity of the thalamus	3	3/35	8.57
Hypodensity of the corpus callosum	2	2/35	5.71
Hypodensity in the cerebellum	2	3/35	8.57
Cerebral hemorrhages/hemorrhagic foci	4	4/35	11.42
Brain swelling and edema	2	2/35	5.71
Brain herniation	1	1/35	2.85
Opacification of paranasal sinuses	1	1/35	2.85
Internal hydrocephalus	1	1/35	2.85
Parenchymal hematoma with surrounding edema	1	1/35	2.85
Cerebral parenchymal volume loss with sulcal enlargement	1	1/35	2.85
Enlargement of the lateral ventricles with intraventricular masses	1	1/35	2.85
Increased supratentorial leptomeningeal enhancement	1	1/35	2.85

### 3.2 Study population

From a total of 45 studies, 53 patients with COVID‐19‐associated encephalitis were enrolled from 18 countries. Forty‐one (93.18%) studies were case reports and 4 (6.82%) were case series. The most significant number of studies was conducted in the USA (*n* = 10), followed by Italy (*n* = 6) and Iran (*n* = 5).

### 3.3 Demographic data

Demographic information of the individuals with COVID‐19‐associated encephalitis can be found in Tables 1 and 2. The patients were 21 female and 32 male with mean age of 52.12 years ranged between 9 months and 89 years. The highest incidence of COVID‐19‐associated encephalitis was observed in people over 50 years of age (54.72%).

### 3.4 Diagnostic methods

COVID‐19 was most often diagnosed by RT‐PCR (92.45%) and chest CT (37.73%). In addition, serological tests (11.32%) and simplexa assay (1.88%) were used to detect SARS‐CoV‐2 virus (Table 3). Brain MRI (81.48%), CSF analysis (46.29%), electroencephalography (42.59%), and head CT (37.03%) were the most frequently used methods to diagnose encephalitis (Table 3). The most common brain MRI patterns were hyperintensity in the white matter (44.68%), hyperintensity in the temporal lobe (17.02%), and hyperintensity of the thalamus (12.76%). In addition, hypodensity of the white matter (17.14%) and cerebral hemorrhages/hemorrhagic foci (11.42%) were the most common head CT scan patterns.

### 3.5 Clinical manifestations

Clinical manifestations were reported in five categories including (A) neurological manifestations such as altered mental status (53.70%), decreased consciousness/unconsciousness (33.33%), and seizure (29.62%); (B) psychiatric symptoms (14.81%); (C) general symptoms such as fever (70.37%), headache (20.37%), weakness/asthenia (18.51%), and drowsiness (16.66%); (D) neuromuscular symptoms such as myalgia (7.40%), myoclonus (5.55%); and (E) other clinical manifestation such as respiratory symptoms (68.51%), renal dysfunction (18.51%), and visual impairment (7.40%).

### 3.6 Comorbidities

The most common comorbidities were hypertension (29.16%), diabetes mellitus (14.58%), obesity (12.50%), and neurologic disorders (10.41%). The less common comorbidities were anemia (2.08%), hypercholesterolemia (2.08%), hypothyroidism (2.08%), vitiligo (2.08%), and asthma (2.08%).

### 3.7 Treatment options

A wide range of treatment options was used to treat COVID‐19. The most common of which were hydroxychloroquine (50%), acyclovir (20%), and ritonavir/lopinavir (16.66%), respectively. Common encephalitis treatment modalities included IV methylprednisolone/oral prednisone (36.11%), IV immunoglobulin (27.77%), acyclovir (16.66%). In Table 3, we summarize all of the drugs used.

### 3.8 Outcomes

In total, 58.69% of the patients with COVID‐19‐associated encephalitis discharged and 13.05% of them were still hospitalized. The pooled mortality rate of these patients was 28.26%.

### 3.9 Risk of bias assessment

The results of the critical appraisal (JBI checklist) of included studies are summarized in Table S1. Overall, 45 articles were identified as having a low risk of bias (quality assessment score >7).

## 4 DISCUSSION

Encephalitis is one of the specific neurological manifestations of COVID‐19 that can cause severe damage to the patient.^16^ In this study, we reviewed case series and case reports to evaluate the clinical symptoms, diagnosis, treatment, and outcome of COVID‐19‐associated encephalitis.

The patients with COVID‐19‐associated encephalitis can show encephalitis weeks after the onset of symptoms of COVID‐19 or to have symptoms of COVID‐19 and encephalitis at the same time.^12^ Our study indicated that the clinical manifestations in patients with COVID‐19‐associated encephalitis can be both central nervous system symptoms (i.e., headache, dizziness, and impaired consciousness) and peripheral nervous system symptoms (i.e., hypogeusia, hyposmia, etc.). The most common symptoms were related to altered mental status (53.7%), decreased consciousness/unconsciousness (33.3%), and seizure (29.6%).

These results were consistent with a systematic review performed by Siow et al. They also reported that decreased level of consciousness (77.1%), alter in mental state (72.3%), and seizures (38.2%) were the most common symptoms in patients with COVID‐19‐associated encephalitis.^12^

Correia et al.^17^ conducted a systematic review on the neurological manifestations of patients with COVID‐19. The rate of altered consciousness in their study was reported to be 11.2%. The difference in the results of their study with us could be due to differences in the time frame of each study and the number of patients admitted.

Furthermore, headache (20.37%) and weakness/asthenia (18.51%) were other clinical symptoms of COVID‐19‐associated encephalitis in the present study. Correia et al.^17^ and Siow et al.^12^ reported headache rates of 16.8% and 27.3%, respectively.

In this study, myalgia (7.4%) was the most frequent neuromuscular symptom. The prevalence of myalgia in a meta‐analysis done by Li et al.^18^ was 35.8%.

Fever (70.37%) and respiratory failure (68.51%) were the most common symptoms of COVID‐19 in our evaluation. Heidary et al.^19^ achieved the same results in their study. They reported that clinical symptoms of COVID‐19 included coughing (81.3%), fever (62.8%), and dyspnea (60%). Also, Koupaei et al.^20^ demonstrated that the COVID‐19 patients mostly suffered from fever (78.8%), cough (63.7%), and respiratory distress (22.6%).

So far, several cases of COVID‐19‐associated encephalitis have been reported in people who did not have symptoms of COVID‐19. The presence of asymptomatic people with encephalitis recommends that performing the diagnostic tests is necessary to prevent the spread of the disease.^21^, ^22^ On the contrary, CNS involvement is similar in the SARS‐CoV‐2, SARS‐CoV, and MERS‐CoV viruses. Thus, it is recommended that more sensitive and specific tests be performed.^23^

In this study, the most common methods used to diagnose encephalitis were MRI (81.48%), CSF analysis (46.29%), electroencephalogram (42.59%), and head CT scan (37.03%). Among the analysis performed on CSF, only 79.41% were positive and showed the presence of viral RNA. This may be due to the mechanism of encephalitis that the virus has not entered CSF and cannot be detected. Moreover, in the early stages of the disease, CSF may have a normal level and cause a false‐negative result.^5^

The most common MRI findings included hyperintensity in the white matter, hyperintensity in the temporal lobe, and hyperintensity in the corpus callosum, respectively. Although the CT findings of patients with COVID‐19‐associated encephalitis usually are not remarkable,^24^ our study showed that the most findings are hypodensity of the white matter (17.14%) and cerebral hemorrhages/hemorrhagic foci (11.42%).

Probably, some of the signs in the imaging are related to the subcortical white matter hyperintensities and microbleeds in the deep gray nuclei caused by underlying diseases.^12^

The association between underlying diseases such as hypertension, diabetes, chronic obstructive pulmonary disease (COPD), cardiovascular disease, and cerebrovascular disease has been identified with COVID‐19. People with the above underlying diseases are more likely than others to develop COVID‐19 and the severity of the disease.^25^ In the present study, patients with COVID‐19‐associated encephalitis had a higher percentage of hypertension (29.16%) and diabetes mellitus (14.58%).

Angiotensin‐converting enzyme 2 (ACE2), the receptor for SARS‐CoV‐2, is abundant in various organs.^3^ Diabetes can increase the serum ACE2. Thus, it is not surprising that diabetes is a common comorbidity in patients with COVID‐19‐associated encephalitis.^26^

In this study, COVID‐19‐associated encephalitis was more common in people over 50 years of age (54.72%). It seems that elderly people with several underlying diseases are less able to physiological rearrangement, which makes them more prone to encephalitis.^27^

Although various treatments have been used to treat COVID‐19‐associated encephalitis, none of them can be used with certainty. At the time of the COVID‐19 epidemic, physicians should suspect SARS‐CoV‐2 as a differentiating factor when certain diseases and neurological symptoms occur.^21^ Our survey showed that IV methylprednisolone/oral prednisone (36.11%), IV immunoglobulin (27.77%), and acyclovir (16.66%) were the common treatment options to treat encephalitis. The healing role of IV immunoglobulin in severe cases of COVID‐19 has been confirmed in several studies.^28^, ^29^, ^30^, ^31^

There are some limitations in this study. First, only case reports and case series were enrolled in this systematic review. Thus, the existence of publication bias should be considered. Second, since our search was limited to articles published in English, some relevant articles in other languages have missed. Third, some studies lacked sufficient data.

## 5 CONCLUSION

In this systematic review, various aspects of COVID‐19‐associated encephalitis including clinical symptoms, diagnosis, treatment, and outcome were studied. COVID‐19‐associated encephalitis is one of the complications of SARS‐CoV‐2, which may accompany with other neurological symptoms and make the patient's condition worse. It usually occur in severe cases and can increase the mortality rate. Thus, it is recommended to pay special attention to neurological symptoms during the COVID‐19 epidemic. Lack of proper attention causes problems such as delay in COVID‐19 diagnosis, virus transmission, and increased mortality. Therefore, further studies on COVID‐19‐associated encephalitis are suggested.

## CONFLICT OF INTEREST

The authors declare that they have no competing interests.

## AUTHORS’ CONTRIBUTION

Maryam Koupaei, Negar Shadabmehr, Mohamad Hosein Mohamadi, Arezoo Asadi, Sajjad Abasi Moghadam, Amirhosein Shekartabar, Mohsen Heidary, and Fazlollah Shokri contributed in revising and final approval of the version to be published. All the authors agreed and confirmed the study for publication.

## Supporting information

Table S1

## Data Availability

All the data in this review are included in the study.
