# Supplementary material for: Clinical symptoms, diagnosis, treatment, and outcome of COVID‐19‐associated encephalitis: A systematic review of case reports and case series
Source: J Clin Lab Anal. 2022 Apr 18;36(5):e24426. doi: 10.1002/jcla.24426 (PMC9102669; doi:10.1002/jcla.24426)
Supplement: Supplementary file 1 — Table S1 [file JCLA-36-e24426-s001.docx]

***Supplement table 1. Quality assessment for validation studies on the clinical symptoms, diagnosis, treatment, and outcome of COVID-19-associated encephalitis***

| First author | Was the sample representative of the target population? | Were study participants recruited in an appropriate way? | Was the sample size adequate? | Were the study subjects and the setting described in detail? | Was the data analysis conducted with sufficient coverage of the identified sample? | Were objective, standard criteria used for the measurement of the condition? | Was the condition measured reliably? | Are all important confounding factors/subgroups/differences identified and accounted for? | Were subpopulations identified using objective criteria? | Was there appropriate statistical analysis? |
| --- | --- | --- | --- | --- | --- | --- | --- | --- | --- | --- |
| Lopes | Yes | No | No | Yes | Yes | Yes | Yes | No | No | Yes |
| Kihira | Yes | Yes | Yes | Yes | Yes | Yes | Yes | Yes | Yes | Yes |
| Barreto-Acevedo | Yes | No | Yes | Yes | Yes | Yes | Yes | Yes | Yes | Yes |
| Delorme | No | No | Yes | Yes | Yes | Yes | Yes | No | Yes | Yes |
| Lopes | No | No | Yes | Yes | Yes | Yes | Yes | No | Yes | Yes |
| Kumar | Yes | Yes | Yes | No | Yes | Yes | Yes | No | No | Yes |
| Novi | Yes | Yes | Yes | No | Yes | Yes | Yes | No | No | Yes |
| Ayuso | Yes | Yes | Yes | Yes | Yes | Yes | Yes | No | No | Yes |
| Khan | Yes | Yes | Yes | Yes | Yes | Yes | Yes | Yes | Yes | Yes |
| Westhoff | Yes | No | No | Yes | Yes | Yes | Yes | No | No | Yes |
| Kamal | Yes | Yes | Yes | Yes | Yes | Yes | Yes | No | No | Yes |
| Rebeiz | Yes | No | Yes | No | Yes | Yes | Yes | No | No | Yes |
| Zoghi | Yes | No | Yes | No | Yes | Yes | Yes | No | No | Yes |
| Moriguchi | Yes | No | No | Yes | Yes | Yes | Yes | No | No | Yes |
| Haqiqi | No | Yes | Yes | Yes | Yes | Yes | Yes | No | No | Yes |
| Pizzanelli | Yes | Yes | Yes | No | Yes | Yes | Yes | No | No | Yes |
| Al Mazrouei | Yes | Yes | Yes | Yes | Yes | Yes | Yes | No | Yes | Yes |
| Sirous | Yes | Yes | Yes | Yes | Yes | Yes | Yes | Yes | Yes | Yes |
| Mardani | No | No | Yes | Yes | Yes | Yes | Yes | No | No | Yes |
| Vandervorst | Yes | Yes | Yes | Yes | Yes | Yes | Yes | No | No | Yes |
| Freire-Álvarez E. | No | No | No | Yes | Yes | Yes | Yes | Yes | Yes | Yes |
| Parsons | Yes | Yes | Yes | No | Yes | Yes | Yes | No | No | Yes |
| Al-olama | Yes | Yes | Yes | Yes | Yes | Yes | Yes | No | No | Yes |
| Goodloe | Yes | No | No | Yes | Yes | Yes | Yes | Yes | Yes | Yes |
| Sattar SBA. | Yes | No | Yes | No | Yes | Yes | Yes | No | No | Yes |
| Haider. | Yes | Yes | No | Yes | Yes | Yes | Yes | Yes | Yes | Yes |
| Cariddi |  |  |  |  |  |  |  |  |  |  |
| Sofijanova | Yes | Yes | Yes | Yes | Yes | Yes | Yes | No | Yes | Yes |
| Ghosh | Yes | Yes | Yes | Yes | Yes | Yes | Yes | Yes | Yes | Yes |
| Pilotto | No | No | Yes | Yes | Yes | Yes | Yes | No | No | Yes |
| Azab | Yes | Yes | Yes | Yes | Yes | Yes | Yes | No | No | Yes |
| Abdi | Yes | No | Yes | Yes | Yes | Yes | Yes | Yes | Yes | Yes |
| Dharsandiya | No | No | Yes | Yes | Yes | Yes | Yes | No | Yes | Yes |
| Babar | No | No | Yes | Yes | Yes | Yes | Yes | No | Yes | Yes |
| Virhammar | Yes | Yes | Yes | No | Yes | Yes | Yes | No | No | Yes |
| Farhadian | Yes | No | Yes | Yes | Yes | Yes | Yes | Yes | Yes | Yes |
| Henriques-Souza | No | No | Yes | Yes | Yes | Yes | Yes | No | Yes | Yes |
| Afshar | No | No | Yes | Yes | Yes | Yes | Yes | No | Yes | Yes |
| Crosta | Yes | Yes | Yes | No | Yes | Yes | Yes | No | No | Yes |
| Sangare | Yes | Yes | Yes | No | Yes | Yes | Yes | No | No | Yes |
| El-Zein | Yes | Yes | Yes | Yes | Yes | Yes | Yes | No | No | Yes |
| Etemadifar | Yes | No | Yes | Yes | Yes | Yes | Yes | Yes | Yes | Yes |
| Peng | No | No | Yes | Yes | Yes | Yes | Yes | No | Yes | Yes |
| Hayashi | No | No | Yes | Yes | Yes | Yes | Yes | No | Yes | Yes |
| Kumar | Yes | Yes | Yes | No | Yes | Yes | Yes | No | No | Yes |
| Muccioli | Yes | No | Yes | Yes | Yes | Yes | Yes | Yes | Yes | Yes |
